# Soluble Levels of CD163, PD-L1, and IL-10 in Renal Cell Carcinoma Patients

**DOI:** 10.3390/diagnostics12020336

**Published:** 2022-01-28

**Authors:** Sabina Davidsson, Sofia Huotilainen, Jessica Carlsson, Pernilla Sundqvist

**Affiliations:** Department of Urology, Faculty of Medicine and Health, Örebro University, 70362 Örebro, Sweden; sofia.huotilainen@gmail.com (S.H.); Jessica.Carlsson@regionorebrolan.se (J.C.); pernilla.sunqvist@regionorebrolan.se (P.S.)

**Keywords:** renal cell carcinoma, liquid biopsy, sPD-L1, sCD163, sIL-10

## Abstract

CD163+ M2 macrophages have been suggested to counteract tumor immunity by increasing immunosuppressive mechanisms including PD-L1 and IL-10 expression. Soluble levels of PD-L1, IL-10, and CD163 have been reported as potential biomarkers in various cancers, although the prognostic value in renal cell carcinoma (RCC) has to be further elucidated. In the present study, we measured the levels of sPD-L1, sIL-10, and sCD163 in 144 blood samples from patients with RCC. The levels were determined by using enzyme linked immunosorbent assays. Soluble PD-L1 and CD163 were detectable in 100% of the serum samples, and sCD163 in 22% of the urine samples, while only a minority of the samples had detectable sIL-10. Significantly higher serum levels of sPD-L1 and sCD163 were observed in patients with metastatic disease (*p* < 0.05). The results also showed that patients with high levels of sPD-L1 in serum had shorter cancer-specific survival compared with patients with low levels (*p* = 0.002). The results indicate that sPD-L1 most significantly reflects tumor progression in RCC.

## 1. Introduction

Renal cell carcinoma (RCC) has the highest mortality rate of all genitourinary cancers, and constitutes approximately 2% of all malignancies. The clinical outcome in RCC patients varies considerably, but the prognosis for patients with localized disease is generally good, with a five-year survival rate of approximately 90% after tumor removal by partial or radical nephrectomy [1]. Unfortunately, the corresponding five-year survival rate in patients with metastatic disease is dramatically lower, less than 15%. Currently, there is a lack of biomarkers for prognosis of RCC, apart from the histological criteria of grading and staging. Finding new biomarkers that clinicians can use as additional tools to predict prognosis or for repeated monitoring would therefore be of great value, preferably identified in blood samples since they are easier and safer to collect.

Extensive data support the hypothesis that the immune system plays an important role in hampering tumor initiation and progression. Thus, evasion of the immune system is now considered as a hallmark of cancer [2]. By creating an immune suppressive tumor microenvironment (TME), constituted by immunosuppressive cells and immunosuppressive mediators, tumors improve their ability to give rise to a clinically relevant cancer. Tumor-associated macrophages (TAMs) comprise a large fraction of the immune cells in the TME. In response to various signals, TAMs can undergo classical (M1) or alternative (M2) activation, which are the extremes of a wide spectrum of polarized activation states that differ in terms of receptors, cytokine expression, and effector function. M1 macrophages are immunostimulatory and anti-tumoral, while M2 macrophages, characterized by the expression of CD163, enhance tumor progression and support metastasis [3]. The molecular mechanisms involved in the immune modulatory effects of M2 macrophages have not been completely elucidated, but it has been proposed that these macrophages suppress the anti-tumor immune response through expression of the inhibitory molecule programmed death ligand 1 (PD-L1) and secretion of the immunosuppressive mediator Interleukin-10 (IL-10) [4]. Since M2 macrophages are the most abundant immune cell in the TME of renal tumors, this mechanism may be used to assist tumor progression in RCC.

Programmed death ligand 1 (PD-L1) is an inhibitory molecule that when interacting with its receptor PD-1, acts as a negative regulator of the T cell-mediated anti-tumor immunity. The prognostic value of membranous PD-L1 expression has been extensively investigated in a number of malignancies, including several urological malignancies such as renal, bladder, prostate, and penile cancer. For the majority of cancer types investigated, high PD-L1 expression was associated with more advanced tumors and poorer prognosis [5,6,7,8,9].

Until now, IL-10 has been considered an immunosuppressive cytokine due to its association with multiple regulatory or suppressive immune cells, including regulatory T-cells, myeloid-derived suppressor cells, and M2 macrophages. In vitro experiments have shown that IL-10 has the capability to impair proliferation and cytotoxic activity of anti-tumor T-cells, thereby stimulating tumor growth [10]. A meta-analysis found that high levels of IL-10 in serum were correlated with adverse survival in patients with different solid tumors [11]. Conversely, other studies have suggested that IL-10 is equally likely to enhance anti-tumor immunity [12].

Previous evaluations of the prognostic value of CD163, PD-L1, and IL-10 have reported associations between increased expression and poor clinical outcome in several different malignancies [7,13,14,15,16,17,18,19,20]. For example, oral squamous cell carcinoma patients overexpressing CD163 had significantly worse overall survival; gastric cancer patients with high infiltration of IL-10 producing macrophages had shorter recurrence-free survival. In addition, a positive association between PD-L1 expressing tumor cells and cancer-specific death has also been reported for RCC patients. However, the majority of investigations concerning the prognostic value in RCC have been performed using tumor tissue. Blood and urine samples are easier to harvest than tissue samples, thus it is of interest to evaluate levels of soluble CD163 (sCD163), PD-L1 (sPD-L1), and IL-10 (sIL-10) with emphasis on prognostic implications.

In the present study, we investigated the feasibility to measure the levels of sCD163, sPD-L1, and sIL-10 in prospectively collected preoperative serum and urine samples from patients diagnosed with RCC. We also assessed associations between the levels and clinical parameters related to worse outcome.

## 2. Results

In this study, we included preoperatively collected serum and urine samples from 181 patients undergoing partial or radical nephrectomy for suspected RCC, where 37 patients were diagnosed with benign tumors (oncocytoma *n* = 24, angiomyolipoma *n* = 6, kidney cyst *n* = 5, leiomyoma *n* = 1, and metanephric adenoma *n* = 1) and 144 patients with malignant tumors. The patients diagnosed with RCC had a median age of 68 years (range: 30–86 years) and a median BMI of 27.1 (range: 21.1–39.9). Their clinical and pathological characteristics are summarized in Table 1. The median follow-up time was 51.8 months (range: 4.0–85.6 months). At the last follow-up, 13 of the 144 patients had died from RCC.

### 2.1. Preoperative Serum and Urine Levels of sCD163, sPD-L1, and sIL-10

When analyzing the serum samples from the total cohort, sCD163 and sPD-L1 were detectable in all samples and sIL-10 in 11.6% (21/181). Similar rates were observed when including only RCC-patients, sCD163 and sPD-L1 were detectable in 100% (144/144) of the samples and sIL-10 in 13.2% (19/144). The median serum levels of sCD163, sPD-L1, and sIL-10 were 741.4 ng/mL (range: 618.9–890.8 ng/mL), 87.2 pg/mL (79.1–96.7 pg/mL), and 0.9 pg/mL (0.9–1.4 pg/mL), respectively. In urine samples, sPD-L1 was detectable in 22.1% (40/181) of the samples. The median urine level of sPD-L1 was 68.2 pg/mL (63.8–91.2 pg/mL). Significantly higher serum levels of sCD163 were observed in patients with malignant tumors compared to patients with benign tumors, 747.4 pg/mL (339.2–2386.7 pg/mL) versus 674.3 pg/mL (303.3–1538.5 pg/mL) (*p* = 0.044). A trend for higher levels in patients with malignant tumors was also observed for sPD-L1 in serum, 87.2 pg/mL (64.9–348.2 pg/mL) versus 80.2 pg/mL (72.5–123.8 pg/mL) (*p* = 0.054). Investigation of the relationship between preoperative serum levels of sCD163 and sPD-L1 in RCC patients showed a low correlation between sCD163 and sPD-L1 (R = 0.296, *p* = 0.001).

There were no significant differences between men and women in serum or urine levels of either sCD163 or sPD-L1 or in urine levels of CD163. Moreover, no significant differences in the levels of sCD163 or sPD-L1 were found between different histologic tumor subtypes, between smokers and non-smokers, or between patients with BMI over or under 30. Due to the low number of RCC patients with detectable sIL-10, this marker was not included in subsequent analyses. In addition, no validated ELISA-kit for measuring levels of sCD163 in urine was available at the time of analyses.

### 2.2. Associations between Preoperative sCD163 and sPD-L1 Levels and Clinico-Pathological Characteristics in RCC

We next assessed the relationship between preoperative levels of sCD163 and sPD-L1 and various clinico-pathological characteristics.

**Figure 1:**
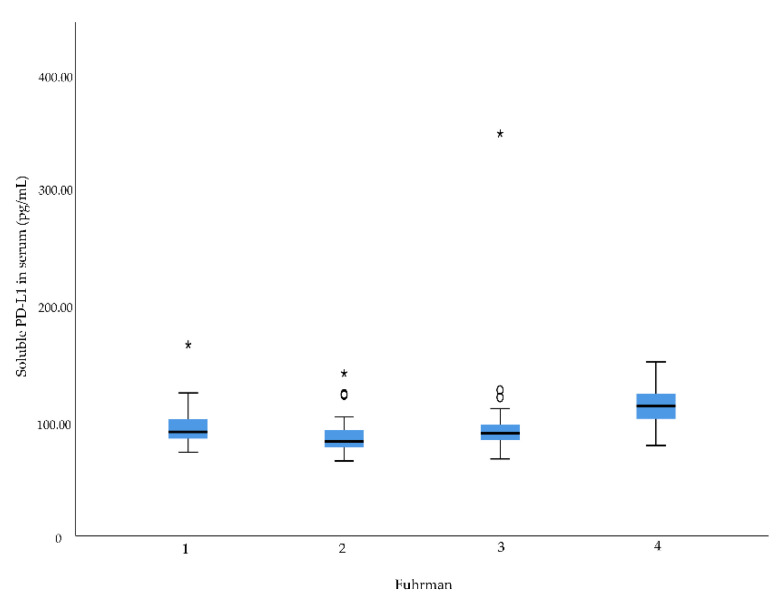
Soluble programmed death ligand 1 (sPD-L1) serum levels in renal cell carcinoma patients with different Fuhrman grades. Higher sPD-L1 level was associated with higher Fuhrman grade, *p* = 0.001. Outliers in the data are represented by a circle (°), and extreme outliers are represented by an asterisk (*).

**Figure 2:**
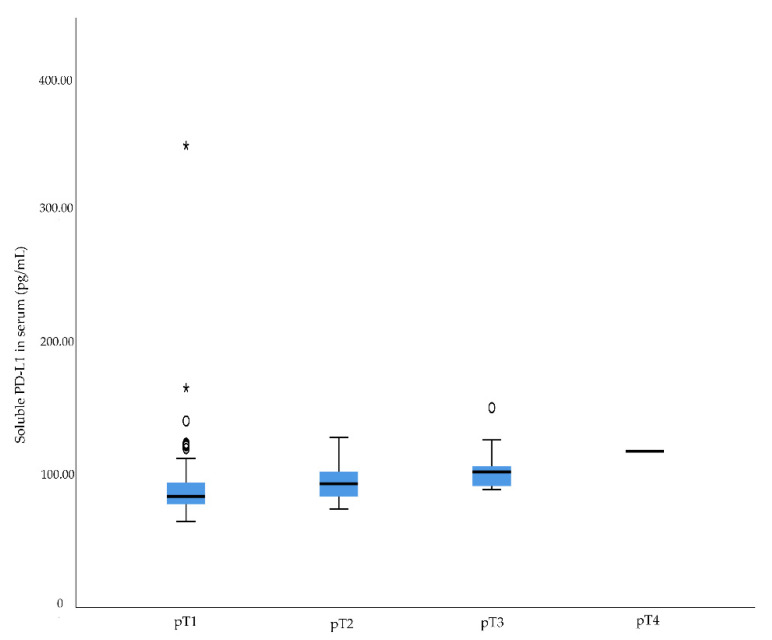
Soluble programmed death ligand 1 (sPD-L1) serum levels in renal cell carcinoma patients with different pT stages. Higher sPD-L1 level was associated with higher pT stage, *p* = 0.001. Outliers in the data are represented by a circle (°), and extreme outliers are represented by an asterisk (*).

There was a significant difference in serum levels of sPD-L1 between Fuhrman grades (*p* = 0.001) and pT stages (*p* = 0.001) (Table 2, Figure 1 and Figure 2). No association was observed between serum levels of sPD-L1 and N stage.

In contrast, preoperative serum levels of sCD163 were not significantly associated with any clinicopathologic characteristics, although a trend was observed between increased serum levels of sCD163 and higher Fuhrman grade (*p* = 0.065) and pT stage (*p* = 0.066) (Table 2).

**Figure 3:**
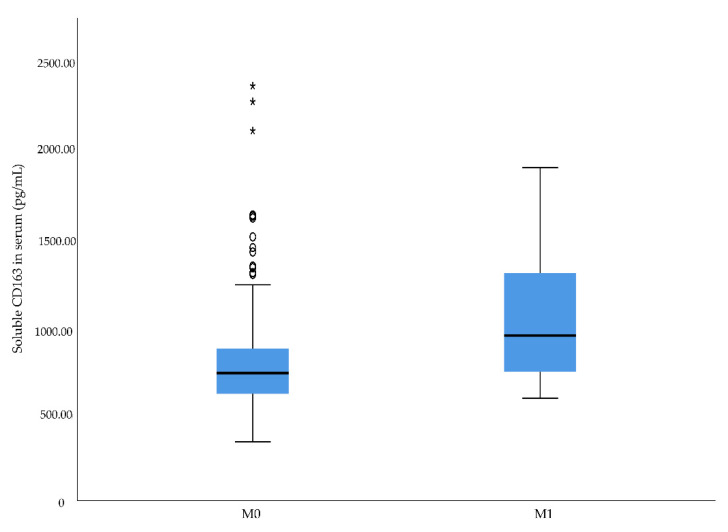
Soluble hemoglobin scavenger receptor (CD163) serum levels in renal cell carcinoma patients with or without metastatic disease at time of diagnosis, *p* = 0.041. Outliers in the data are represented by a circle (°), and extreme outliers are represented by an asterisk (*).

**Figure 4:**
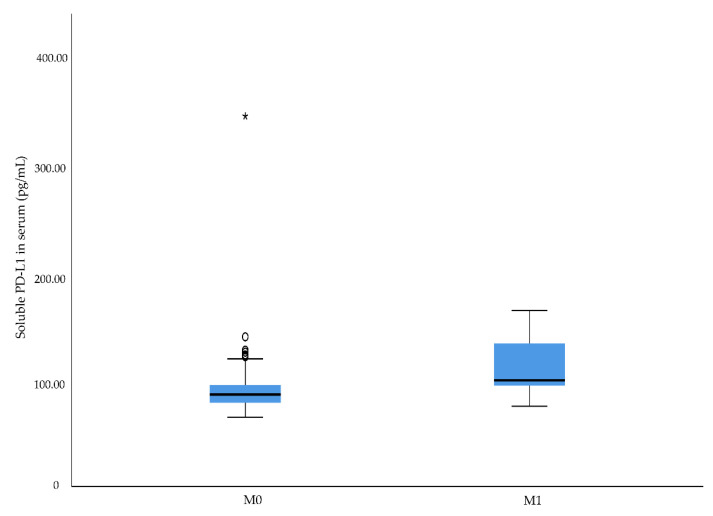
Soluble programmed death ligand 1 (sPD-L1) serum median levels in renal cell carcinoma patients with or without metastatic disease at time of diagnosis, *p* = 0.011. Outliers in the data are represented by a circle (°), and extreme outliers are represented by an asterisk (*).

No significant associations were found between urine sPD-L1 levels and clinico-pathological characteristics. However, an increased level was seen in patients with pT3 tumors compared to patients with pT1 tumors: 65.6 pg/mL (63.2–77.7 pg/mL) versus 99.4 pg/mL (99.4–99.4 pg/mL). To analyze possible associations between preoperative levels of sCD163 and sPD-L1 and aggressive RCC, we assessed the relationship in respect of metastatic disease at diagnosis. The results showed that RCC patients with metastatic disease at diagnosis had significantly higher serum sCD163 levels than patients presenting without metastatic disease (*p* = 0.041) (Table 2 and Figure 3). A significant association was also found between metastatic disease at diagnosed and higher levels of serum sPD-L1 (*p* = 0.011) (Table 2 and Figure 4). The data also revealed that patients with metastatic RCC had significantly higher sPD-L1 levels in urine compared to patients without metastases: 72.4 pg/mL (69.1–72.4 pg/mL) versus 67.2 pg/mL (63.7–87.9 pg/mL) (*p* = 0.023) (Table 2). However, sPD-L1 was only detectable in urine samples of approximately 20% of the patients.

### 2.3. Associations between Preoperative sCD163 and sPD-L1 Levels and Cancer-Specific Death

In the next step, associations between the median levels of sCD163 and sPD-L1 and RCC-related death were evaluated. A negative association was observed between sPD-L1 serum levels and RCC-specific death (*p* = 0.001) (Table 2), where the median sPD-L1 was significantly higher in patients who died from RCC during follow-up compared to patients who survived (100.1 pg/mL versus 84.9 pg/mL). The analyses revealed no significant difference in levels of sCD163 in serum and RCC-specific death (*p* = 0.219), or between sPD-L1 levels in urine and RCC-specific death (*p* = 0.115) (Table 2).

**Figure 5:**
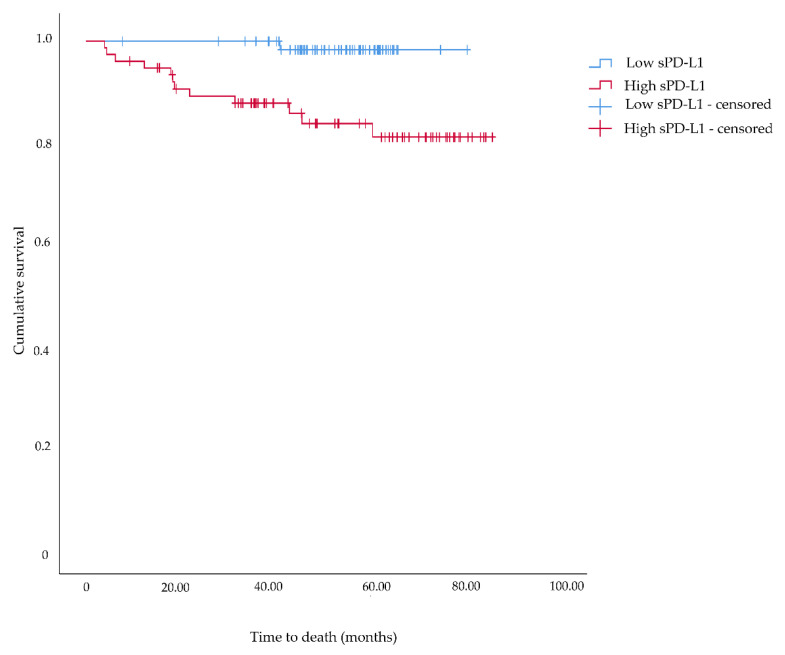
Kaplan–Meier survival plot for association of serum levels of soluble programmed death ligand 1 (sPD-L1) and cancer-specific survival among patients diagnoses with renal cell carcinoma.

The median follow-up time of the patients was 51.8 months, and 13 patients died from RCC during this period. According to the Kaplan–Meier analysis, patients with high levels of sPD-L1 in serum had significantly shorter cancer-specific survival than those with low levels of sPD-L1: 18.5 months versus 40.9 months (*p* = 0.002) (Figure 5). However, a Cox regression model adjusting for pT stage, M stage, Fuhrman grade, and tumor size revealed no increased hazard of dying from RCC (HR: 4.03; 95% CI: 0.25–65.37).

## 3. Discussion

Renal tumors evade eradication by the immune system through a number of mechanisms, including creating an immunosuppressive TME comprising increased infiltration of immunosuppressive cells and increased expression of immune inhibitory mediators. In the present study, we investigated the feasibility to measure soluble levels of CD163, PD-L1, and IL-10 in blood samples from 144 RCC patients. We also investigated associations between the levels and clinical parameters related to adverse outcome. Soluble PD-L1 and sCD163 were detectable in all serum samples while sIL-10 was detectable in only a minority of the samples. In urine, approximately 20% of the patients had detectable levels of sPD-L1. The results further showed a positive association between sPD-L1 and adverse clinical outcome, especially sPD-L1 in serum, and it appears the urine levels of sPD-L1 are higher in RCC patients presenting with metastatic disease at diagnosis.

Tumor-associated macrophages represent a major part of the leukocyte infiltrate in the TME, where the dominant phenotype is CD163+ M2 macrophages with tumor-promoting functions. Among other things, these macrophages have the capability to produce immunosuppressive cytokines such as IL-10 and express immune response inhibitory molecules such as PD-L1. Analysis performed on TAMs isolated from RCC tumors revealed frequent infiltration of CD163+ M2 macrophages associated with increased production of IL-10 and enhanced expression of PD-L1. A substantial number of investigations have reported high infiltration of tumoral CD163+ M2 macrophages and increased PD-L1 expression as independent prognostic factors for poor clinical outcome in various malignancies, including RCC. However, measurement of tissue-based prognostic markers requires an invasive sample procedure, thus finding prognostic markers demanding a less invasive sampling technique is preferable. Given the relationship between the abovementioned markers and poor prognosis, it may be reasonable to speculate that the soluble level of each marker would also be of prognostic significance in RCC patients. In the present study, a low positive correlation was observed between levels of sCD163 and sPD-L1, suggesting that both CD163-mediated and PD-L1-mediated pathways contribute to immunosuppression in RCC. A significant correlation between serum levels of sCD163 and sPD-L1 was also observed in patients diagnosed with hepatocellular carcinoma [21,22].

High serum levels of sPD-L1 are related to poor prognosis in various cancers, such as hepatocellular carcinoma, esophageal cancer, lung cancer, gastric cancer, rectal cancer, and lymphoma [23]. In line with these reports, our data showed that high serum levels of sPD-L1 were associated with well-established adverse clinico-pathological characteristics also in RCC. Patients with high levels of sPD-L1 more commonly presented with tumors with a higher stage and grade as well as tumors with metastases at distant locations. Similar findings have been reported previously in three independent studies, evaluating serum levels of sPD-L1 in preoperatively collected serum samples from RCC-patients. In conclusion, they found that elevated levels were associated with less differentiated tumors with higher invasive and metastatic potential [24,25,26]. In the present study, we also revealed that RCC patients with high serum levels of sPD-L1 had a shorter survival time compared to patients with low levels, which adds further support for a potential value of sPD-L1 as an additional tool in future prognostic implication in RCC. Although we were not able to identify sPD-L1 as an independent biomarker for survival, Fukuda et al. and Larrinaga et al. reported sPD-L1 as an independent biomarker for shorter overall survival and shorter 5-year overall survival for RCC patients [26,27]. The data, all together, encourage further evaluation to elucidate the significance of sPD-L1 in serum as an additional prognostic tool in RCC. It should, however, be noted that conflicting data concerning the prognostic value of sPD-L1 in RCC also exist. For instance, no relation to more aggressive tumors, recurrence, or survival were found in a recent evaluation of the prognostic value of 14 different soluble immune checkpoint proteins (including sPD-L1) in plasma samples from ccRCC patients [28]. It should, however, be highlighted that the latter study measured sPD-L1 in plasma compared to previous study that measured sPD-L1 in serum.

A recent proof-of concept study provided data showing that sPD-L1 can also be detected in urine samples. Patients with urinary bladder cancer were found to have significantly higher urinary levels of sPD-L1 compared to patients with non-malignant urological diseases [29]. The present study, to the best of our knowledge, is the first to evaluate the detection rate of sPD-L1 in urine from patients with RCC and further investigate relations between levels and clinic-pathological characteristics and survival. Soluble PD-L1 was detectable in approximately a fifth of the samples with equally high levels in men and women with RCC, in different histological tumor subtypes, in smokers and non-smokers, and in patients with BMI over or under 30. No associations between urinary levels of sPD-L1 and Fuhrman grade or pT stage were found, although it is noteworthy that higher levels were observed in RCC patients with pT3 tumors compared to patients with pT1 tumors. Interestingly, higher levels were also more common in patients presenting distant metastasis at time of diagnosis. Based on these findings, and due to the easy sampling, further studies should assess the value of sPD-L1 in urine in RCC even though only 22% of the samples had detectable sPD-L1 in the present cohort.

Increased serum levels of sCD163 have been reported previously as an independent marker for predicting poor prognosis in patients diagnosed with B-cell chronic lymphocytic leukemia, myeloma, melanoma [30,31,32]. High serum levels have also been observed in gastric cancer patients with aggressive tumors and short survival time [33]. In addition, high serum levels of sCD163 were reported as an independent predictor of shorter survival in hepatocellular carcinoma patients [22], and associated with poor prognosis in terms of shorter disease-free survival among patients with ovarian cancer [32]. To the best of our knowledge, no previous study has evaluated the relations between serum levels of sCD163 and clinico-pathological characteristics in RCC. Although no clear associations were observed between sCD163 and Fuhrman grade or pT stage, patients presenting with distant metastasis at diagnosis had significantly higher levels of sCD163. Future studies are needed to further evaluate the clinical value of serum sCD163, building upon the initial data presented here. Further, it would be of interest to study sCD163 and sPD-L1 in clinical settings, as additional prognostic tools in regard to follow-up schemes or as tools for monitoring patients with metastatic RCC undergoing targeted and/or immunotherapies.

Previous investigations of sIL-10 as a prognostic marker in RCC have produced limited data with inconclusive results. Wittke et al. detected high levels of sIL-10 in 25% of the serum samples collected from 80 patients diagnosed with metastatic renal carcinoma, and found that a high level was an independent predictor of unfavorable clinical outcome [34]. On the other hand, Guida et al. found no association between sIL-10 levels and adverse prognosis in patients with metastatic RCC [35]. In the present study, sIL-10 was only detectable in a small proportion of the blood samples, leaving us with no power for further investigation.

Since different histological subtypes in RCC have different prognoses, we also performed a subgroup analyses including only patients diagnosed with clear cell RCC, revealing the same results as when including patients with all histological subtypes (Data not shown).

This study has some limitations. The relatively small number of participants and outcome and the short follow-up period limited the possibility to draw definite conclusions concerning the value of sPD-L1 and sCD163 as predictive biomarkers for shorter cancer-free survival in RCC. However, the data presented here motivate further evaluations using larger cohorts with longer follow-up periods. It can also be viewed as a limitation that we did not evaluated correlations between sPD-L1 and sCD163 and PD-L1- and CD163- expression in tumor tissue from the patients.

## 4. Materials and Methods

Between February 2014 and November 2017, patients were recruited to the Renal Cancer Blood Urine Study (REBUS), a prospective cohort study in which all patients referred to the Department of Urology, Örebro University Hospital, Sweden, with suspected RCC planned for surgery were asked to participate. On the day of study inclusion, demographic data were collected from each patient as well as preoperative blood and urine samples. All patients underwent partial or radical nephrectomy according to current guidelines. Tumor grade and clinical stage were determined according to the Fuhrman system and the TNM classification, respectively. The study cohort was followed for cancer-specific mortality until March 2021.

### 4.1. Measurement of sCD163, sPD-L1, and sIL-10 in Serum and Urine Samples

Blood samples were allowed to clot for 30 min at room temperature and serum was obtained by centrifugation at 3000× *g* for 10 min at 4 °C. The urine samples were centrifuged at 2000 rpm for 10 min. After centrifugation, both serum and urine samples were aliquoted and stored at −80 °C until further analyses.

Soluble CD163 in serum was measured using a commercially available human CD163 enzyme-linked immunosorbent assay (ELISA) according to the manufacturer’s instructions (R&D Systems Inc., Minneapolis, MN, USA, catalog no. DC1630). The detection range was 1.56–100 ng/mL. Each sample was analyzed in duplicate along with protein standards and controls with known concentrations (low, medium, and high; R&D Systems Inc., catalog no. QC261). The optical density was measured using a Multiskan Ascent plate reader (Thermo Fisher Scientific, Waltham, MA, USA) at 450 and 540 nm. The coefficient of variation (CV, %) was calculated for each sample, and all samples with a value above 10% were analyzed a second time. The mean values of absorbance versus concentration were plotted and a log/log curve-fit was applied.

Soluble PD-L1 in serum and urine was measured using a commercially available human PD-L1 ELISA according to the manufacturer’s instructions (R&D Systems Inc., catalog no. DB7H10). The detection range was 25–1600 pg/mL. Each sample was analyzed in duplicate along with protein standards and controls with known concentrations (low, medium, and high; R&D Systems Inc., catalog no. QC226). The optical density was measured using a Multiskan Ascent plate reader (Thermo Fisher Scientific) at 450 and 540 nm. The CV was calculated for each sample, and all samples with a value above 10% were analyzed a second time. The mean values of absorbance versus concentration were plotted, and a four-parameter logistic non-linear regression model fit was applied.

Soluble IL-10 in serum was measured using a commercially available human IL-10 ELISA according to the manufacturer’s instructions (R&D Systems Inc., catalog no. HS100C). The detection range was 0.78–50 pg/mL. Each sample was analyzed in duplicate along with protein standards and controls with known concentrations (low, medium, and high; R&D Systems Inc., catalog no. QC41). The optical density was measured using a Multiskan Ascent plate reader (Thermo Fisher Scientific) at 490 and 650 nm. The CV was calculated for each sample, and all samples with a value above 10% were analyzed a second time. The mean values of absorbance versus concentration were plotted, and a four-parameter logistic non-linear regression model fit was applied.

All samples were analyzed retrospectively, and the technician performing the CD163, PD-L1, and IL-10 measurements was blinded to the pathological features and outcome of the patients analyzed.

All patients were informed about the study before giving written consent. The study was approved by the Ethical Review Board in Uppsala-Örebro, Sweden (2012/161).

### 4.2. Statistics

In order to test the data for normality, a Shapiro-Wilks test was used. A Spearman’s correlation test was used to investigate correlations between the two soluble markers. Differences in levels of sCD163 and sPD-L1 between groups were determined by using the non-parametric Wilcoxon–Mann–Whitney and Kruskal-Wallis tests. Box plots were created in which the horizontal boundaries of the boxes represented the first and third quartile and the vertical line in between indicated the median. The Kaplan–Meier method was used to estimate survival probabilities and the log-rank test was used to compare groups. The data were dichotomized as high or low (over and under median). A Cox regression model was used to estimate hazard ratios (HRs) and 95% confidence intervals (CIs). All analyses were performed in SPSS version 22.0 (IBM SPSS, New York, NY, USA). Statistical significance was set at *p* < 0.05.

## 5. Conclusions

In the present study, sPD-L1 and sCD163 were detectable in all 144 serum samples analyzed as opposed to sIL-10, which was detectable in only a minority of the samples. Soluble PD-L1 most significantly reflected tumor progression, since patients with high levels of sPD-L1 presented metastatic disease at diagnosis and had shorter cancer-specific survival. Identifying new biomarkers to predict outcomes and for therapeutic monitoring of RCC patients are urgently needed. Using soluble biomarkers would be preferable, since sampling a blood sample is less invasive than collecting a tissue biopsy. Additional studies in larger cohorts with longer follow-up periods are needed to further investigate the role of sPD-L1 and sCD163 as prognostic biomarkers in RCC.

## Figures and Tables

**Table 1:** Clinical and pathological characteristics of patients with renal cell carcinoma (RCC).

	RCC Cases, *n* = 144
Subtypes of RCC, *n* (%)	
Clear cell RCC	110 (76.4)
Chromophobe RCC	11 (7.6)
Papillary RCC type I	18 (12.5)
Papillary RCC type II	5 (3.5)
Tumor diameter, mm (range)	50.5 (13–230)
pT stage, *n* (%)	
1	115 (79.9)
2	18 (12.5)
3	10 (6.9)
4	1 (0.7)
pN stage, *n* (%)	
0	142 (98.6)
1	2 (1.4)
M stage, *n* (%)	
0	136 (94.4)
1	8 (5.6)
Fuhrman, *n* (%)	
1	24 (17.1)
2	66 (47.2)
3	42 (30.0)
4	8 (5.7)
Missing	4

**Table 2:** Associations between median levels of soluble PD-L1 and CD163 in serum and urine and clinico-pathological characteristics in patients with renal cell carcinoma.

Variable	sPD-L1 Serum	*p*-Value	sCD163 Serum	*p*-Value	sPD-L1 Urine	*p*-Value
Fuhrman grade		0.001		0.065		0.559
1	90.0		731.4		68.1	
2	81.8		740.3		66.3	
3	89.0		698.0		68.9	
4	112.2		1378.4		92.4	
pT stage		0.001		0.066		0.136
1	83.8		731.5		65.6	
2	93.3		941.1		69.1	
3	102.1		757.4		99.4	
4	117.8		1916.9			
pN stage		0.993		0.215		0.763
0	87.2		739.2		68.2	
1	108.6		990.0			
M stage		0.011		0.041		0.023
0	86.3		734.3		67.2	
1	99.8		950.7		72.4	
Cancer-specific death		0.001		0.219		0.115
No	85.0		736.8		67.2	
Yes	100.1		799.0		91.2	

## Data Availability

The data presented in this study are available on request from the corresponding author. The data are not publicly available due to ethical restrictions.
